# Cohort profile: the Taiwan Initiative for Geriatric Epidemiological Research - a prospective cohort study on cognition

**DOI:** 10.4178/epih.e2024057

**Published:** 2024-06-25

**Authors:** Pei-Iun Hsieh, Te-Hsuan Huang, Jeng-Min Chiou, Jen-Hau Chen, Yen-Ching Chen

**Affiliations:** 1 Institute of Epidemiology and Preventive Medicine, College of Public Health, National Taiwan University, Taipei, Taiwan; 2 Institute of Statistics and Data Science, National Taiwan University, Taipei, Taiwan; 3 Institute of Statistical Science, Academia Sinica, Taipei, Taiwan; 4 Department of Geriatrics and Gerontology, National Taiwan University Hospital, Taipei, Taiwan; 5 Department of Internal Medicine, National Taiwan University College of Medicine, Taipei, Taiwan; 6 Department of Public Health, College of Public Health, National Taiwan University, Taipei, Taiwan

**Keywords:** Cognition, Cohort studies, Aged

## Abstract

The Taiwan Initiative for Geriatric Epidemiological Research (TIGER) was founded in 2011 to elucidate the interrelationships among various predictors of global and domain-specific cognitive impairment, with the aim of identifying older adults with an increased risk of dementia in the preclinical phase. TIGER, a population-based prospective cohort, recruited 605 and 629 (total of 1,234) older adults (aged 65 and above) at baseline (2011-2013 and 2019-2022) of phase I and II, respectively. Participants have undergone structured questionnaires, global and domain-specific cognitive assessments, physical exams, and biological specimen collections at baseline and biennial follow-ups to date. By 2022, TIGER I has included 4 biennial follow-ups, with the participants comprising 53.9% female and having a mean age of 73.2 years at baseline. After an 8-year follow-up, the annual attrition rate was 6.1%, reflecting a combination of 9.9% of participants who passed away and 36.2% who dropped out. TIGER has published novel and multidisciplinary research on cognitive-related outcomes in older adults, including environmental exposures (indoor and ambient air pollution), multimorbidity, sarcopenia, frailty, biomarkers (brain and retinal images, renal and inflammatory markers), and diet. TIGER’s meticulous design, multidisciplinary data, and novel findings elucidate the complex etiology of cognitive impairment and frailty, offering valuable insights into factors that can be used to predict and prevent dementia in the preclinical phase.

## INTRODUCTION

In March 2018, Taiwan officially became an aged society, with more than 14% of the population being older adults. As the population ages, dementia, a common geriatric syndrome, has become a critical public health concern for older adults. Alzheimer’s disease (AD), the most common cause of dementia, is characterized by cognitive and behavioral impairment that hinders daily life [1] and currently lacks effective treatment. According to the World Health Organization, more than 55 million people have been diagnosed with dementia, making it the seventh leading cause of death worldwide [2]. A survey of Taiwanese older adults (2011-2013) found an age-adjusted and sex-adjusted prevalence of 8.1% for all-cause dementia and 18.8% for mild cognitive impairment (MCI) [3]. In 2019, the number of cases attributed to all-cause dementia in Taiwan reached 0.2 million based on the National Health Insurance Database operated by the Ministry of Health and Welfare [4]. Therefore, identifying and preventing cognitive impairment before clinical manifestation is crucial.

Numerous prospective cohort studies have been established to investigate factors associated with cognitive impairment or dementia, including socio-demographics, lifestyle, environmental exposure, clinical factors, and biomarkers [5]. However, these factors vary across countries, geographic locations, and ethnic groups, and some large cohorts worldwide lack repeated measures of environmental and clinical factors (e.g., retinal biomarkers and oral examinations) [6-9]. In Taiwan, some population-based cohort studies primarily recruiting older adults have been conducted, including the Taiwan Longitudinal Study on Aging (TLSA) [10], Healthy Aging Longitudinal Study in Taiwan (HALST) [11], I-Lan Longitudinal Aging Study (ILAS) [12], and Taichung Community Health Study for Elders (TCHS-E) [13]. However, limited studies have explored multidisciplinary factors related to cognitive impairment in the preclinical phase of dementia. Additionally, only one Taiwanese cohort study, including middle-aged and older adults, collected domain-specific cognition data [12], and most studies lack regular follow-ups. Furthermore, the cognitive assessments used in some cohorts (e.g., the Mini-Mental State Exam and short Portable Mental Status Questionnaire) are not suitable for community-dwelling older adults because they have difficulty differentiating between MCI and normal cognition. Our study adopts the Montreal Cognitive Assessment (MoCA) from 2011 as it is more challenging and sensitive to small cognitive changes, enabling us to differentiate people with MCI and normal cognition [14,15]. Furthermore, although a large cross-sectional survey was conducted in Taiwan during 2011-2013 [3], providing the prevalence of MCI and all-cause dementia in Taiwanese older adults, no follow-up survey has been conducted since then. As the cognition and various health conditions of older adults tend to change over time, it is crucial to conduct a prospective cohort study with regular follow-ups that collects data on multidisciplinary factors from older adults to capture temporal changes in their health status.

Previous studies in Taiwan have mainly collected cross-sectional data on cognitive impairment [10-12,16], with limited cohort studies [16,17]. Furthermore, longitudinal data on environmental and clinical factors, as well as assessments of global and cognitive domains are especially lacking in some large cohorts worldwide. Therefore, the Taiwan Initiative for Geriatric Epidemiological Research (TIGER), a prospective cohort study, started recruiting community-dwelling older adults without dementia in 2011, with biennial follow-ups since then. TIGER has established collaborations with neurologists, geriatricians, other clinicians in various specialties, biostatisticians, epidemiologists, and experts from several other fields to collect multidisciplinary data, including lifestyle, clinical imaging (e.g., brain magnetic resonance imaging [MRI], retinal optical coherence tomography [OCT] images, and fundus photos), nutritional status (e.g., food frequency questionnaire [FFQ] and serum nutritional biomarkers), environmental exposure (e.g., air pollutants and environmental tobacco smoke), genetic factors (e.g., genetic polymorphisms in the apolipoprotein E [*APOE*] gene), and other biomarkers (e.g., serum metabolomic and inflammatory markers, hair cortisol level, and urine). The primary objective of TIGER is to elucidate the interrelationships among various predictors of global and domain-specific cognitive impairment, aiming to identify older adults with an increased risk of dementia in the preclinical phase. In addition to cognitive function, TIGER includes data on frailty, enabling the prediction of cognitive frailty and subsequent mortality. The longitudinal and multidisciplinary data collected through TIGER have already contributed to novel findings and will continue to do so, reinforcing the development of further research and strategies for the early prediction and prevention of dementia.

The purpose of this work is to provide the cohort profile of TIGER and summarize its critical findings. We hope to contribute to the broader understanding of the multifaceted factors related to cognitive impairment and the early identification and prevention of dementia.

## STUDY PARTICIPANTS

### Study design and population

**Figure 1.**
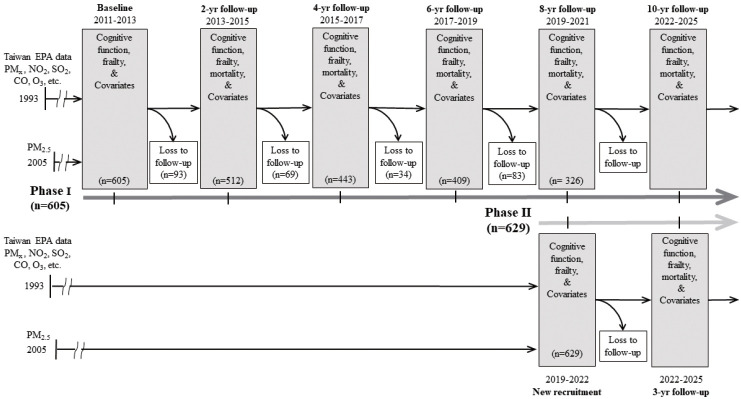
A conceptual timeline of Taiwan Initiative for Geriatric Epidemiological Research (TIGER; 2011 to present; n=1,234) Covariates included the questionnaire variables, and the measurements of physical, biochemical, and genetic. EPA, Environmental Protection Administration; PM, particulate matter; PM_2.5_, particulate matter with a diameter less than 2.5 μm; SO_2_, sulfur dioxide; CO, carbon monoxide; NO_2_, nitrogen dioxide; O_3_, ozone.

TIGER is a prospective cohort study that aims to investigate the predictors and trajectories of cognitive impairment in community-dwelling older adults (aged ≥ 65, n=1,234). In phase I, we recruited 605 participants from the senior health check-up program of the National Taiwan University Hospital (NTUH) during the baseline period of 2011-2013. The participants have been followed biennially since then (2013-2015, 2015-2017, 2017-2019), except for the 2019-2022 wave, which was extended 1 year due to the coronavirus disease 2019 pandemic (Figure 1). In TIGER II, we recruited 629 participants at baseline (2019-2022) with the same follow-up regime.

### Characteristics of the study population

The TIGER study included 605 older adults at baseline (2011-2013), with a mean age of 73.2 years (standard deviation: 5.5). Across age groups (i.e., 65-69, 70-74, 75-79, and ≥ 80), significant differences were observed in the proportion of female, years of education, annual disposable income, physical activity, gait speed, instrumental activities of daily living (IADL), hypertension, and cognitive test scores (Table 1). Table 1 includes only TIGER I information, as the key findings are mainly from this phase.

### Attrition rate

In TIGER I, out of the 605 participants at baseline (2011-2013), 53.8% (n=326) remained in the cohort at the 8-year follow-up (2019-2022), 9.9% (n=60) had passed away by December 2021, and 36.2% (n=219) had dropped out due to illness, poor functional status, or being too busy to participate. There was an average annual attrition rate of 6.1% between 2011 and 2019. To address this concern, we introduced 2 key strategies: first, the inclusion of an additional 629 participants in 2019 (i.e., phase II of TIGER; Figure 1) to maintain the sample size of the cohort, and second, considering the effect of informative dropouts on the outcomes through statistical analyses [18]. These strategies are crucial for mitigating and clarifying the impact of attrition on the integrity of the study. With its well-designed cohort, continuous biennial follow-up, and expanded sample size, we anticipate that TIGER will generate further critical research findings for epidemiological studies on dementia and contribute to our understanding of aging and aged populations.

### Ethics statement

This study was approved by the NTUH Research Ethics Committee (201101039RB, 201112047RIB, 201412213RINC, IRB 201712220RIN, 202012214RIN, 202112042RINA; 201312156RINC, 201712218RIN, 201812102RIN, 202012285RIN, 202112042RINA, and 202312052RINC). All participants provided written informed consent following the Declaration of Helsinki before participating in this project. The research protocol, informed consent, questionnaires, and application forms have been approved by the research ethics committee of NTUH, and all participants have provided written informed consent before joining the study.

## MEASUREMENTS

Estimates and variables related to cognitive impairment collected in TIGER are summarized in Table 2. Longitudinal data were administered by trained interviewers from participants at baseline and each follow-up, including measures of cognitive function, questionnaires, physical measures, environmental exposure, imaging data, and biological specimens, as detailed below.

### Cognitive assessment

A battery of neuropsychological tests was used to assess global and domain-specific cognition, including memory, attention, executive function, and verbal fluency at baseline and each follow-up. Global cognition was evaluated using the Taiwanese version of the Montreal Cognitive Assessment (MoCA-T), a screening tool for MCI (with a sensitivity of 0.92 and specificity of 0.78 validated in a Taiwanese population [19]). The MoCA-T score ranged from 0 to 30, with a score of ≥ 24 indicating normal cognition and < 24 indicating cognitive impairment [19]. Cognitive impairment was further grouped into MCI and suspected dementia. MCI was defined as a MoCA-T score of 22 or 23 with an intact ability to perform IADL. Suspected dementia was determined by a MoCA-T score ≤ 21 with IADL dependency [20]. Episodic memory was assessed using the logical memory-immediate and delayed theme and free recall tests in the third edition of the Wechsler Memory Scale. Attention performance was measured using the Digit Span Forward and Backward tests, with the latter also used to assess working memory. The Trail Making Test A (TMT-A) was used to evaluate attention and executive function, while the TMT-B was used to evaluate working memory and executive function. Verbal fluency tests were used to assess language function, particularly category fluency. Subjective cognitive decline was evaluated in the 8-year follow-up by asking, “Do you have cognitive difficulties compared to the previous year?” and “In the past year, have you experienced cognitive difficulties compared to others of your same age?” [21].

### Imaging data

High-resolution T1-weighted volumetric MRI scans of the brain were performed using a 1.5-T scanner [22] at baseline. The MRI images were processed using the FreeSurfer suite, version 5.3. The average cerebral cortical thickness was estimated for the whole brain and some areas (frontal, parietal, temporal, occipital, limbic, and insular lobar). Additionally, the AD signature area was calculated by averaging the cortical regions including entorhinal, inferior temporal, middle temporal, temporal pole, superior parietal, inferior parietal, posterior cingulate, and precuneus [23]. OCT is used to acquire retinal biomarkers at 4-year and 10-year follow-ups of TIGER I, including retinal nerve fiber layer thickness and the ganglion cell–inner plexiform layer (GC-IPL), and fundus photography is used to collect data for estimating the vascular fractal dimension, which represents the complexity of the retinal vasculature [24,25].

### Physical measures

Physical function measures are obtained through a variety of clinical examinations. Body composition, including body fat and muscle mass, is assessed using bioelectrical impedance analysis. Hand grip strength, which measures muscular strength in the hands and forearms, is evaluated using a dynamometer. The Short Physical Performance Battery, which includes 3 parts (balance test, gait speed, and repeated chair stand), is used to evaluate lower extremity function. The ankle-brachial index, which is the ratio of systolic blood pressure measured at the ankle to that measured at the brachial artery, is used to evaluate arterial perfusion in the lower extremities [26] at baseline and the 6-year follow-up. Carotid intima-media thickness was used to measure the thickness of the vessel wall at baseline using a color-coded ultrasound machine (iE33; Philips Medical Systems, Amsterdam, the Netherlands) with a 3 to 11-MHz linear-array transducer for the extra cranial arteries. Olfactory function is assessed using the Sniffin’ Sticks Identification Test to evaluate odor identification ability [27] at the 4-year and 8-year follow-ups and thereafter. Finally, oral health conditions, including periodontal status, tooth defects, and dentition, are evaluated through oral examinations conducted by dentists at baseline, 6-year follow-up, and thereafter.

### Clinical measures and other covariates

TIGER collects information on several clinical measures and covariates. The medical history of each participant included more than 20 conditions and medication use. Physical activities are assessed using a short version of the International Physical Activity Questionnaire. The participants’ functional status is evaluated using the Barthel index for activities of daily living and IADL. Depressive symptoms are assessed using the Center for Epidemiologic Studies Depression Scale (CES-D). Sleep quality and disturbances during the past month are evaluated using the Pittsburgh Sleep Quality Index (PSQI). Daytime sleepiness is evaluated using the Epworth Sleepiness Scale, which measures the likelihood of a person dozing off during the day in various situations. Finally, TIGER uses a 44-item semi-quantitative FFQ, which is a shortened version of a validated 64-item FFQ for the Taiwanese population, to assess participants’ dietary intake in the previous year [28].

### Environmental exposure

Data on ambient air pollutants are collected from 29 monitoring stations located in Taipei, Keelung, and Taoyuan Cities, through the Taiwan Air Quality Monitoring Network established by the Taiwan Environmental Protective Administration (EPA) from 1994 to the present (https://www.epa.gov.tw/ENG/). Bayesian Maximum Entropy is used to estimate the spatiotemporal distribution of 6 air pollutants (particulate matter with a diameter less than 2.5 μm [PM_2.5_], particulate matter with a diameter less than 10 μm [PM_10_], sulfur dioxide [SO_2_], carbon monoxide [CO], nitrogen dioxide [NO_2_], and ozone [O_3_]) and the individual’s residential exposure to these pollutants [29]. Additionally, TIGER assesses indoor air quality by examining daily indoor time (in hours) and ventilation status defined by window/door openness and use of air-circulating equipment.

### Biological specimens

In addition to the blood data (high- and low-density lipoprotein, and creatinine) and urinary protein from the senior health check-up program at NTUH, we additionally collect blood samples from each participant to determine the concentration of high-sensitivity C-reactive protein, homocysteine, vitamin B12, folate, triglyceride, total cholesterol, tumor necrosis factor-alpha, and cystatin C. Metabolomic profiles are determined at baseline through a nuclear magnetic resonance (NMR) spectroscopy and analyzed by the Chenomx NMR Suite software (Chenomx Inc., Edmonton, AB, Canada), based on chemical shifts (ppm) and the multiplicity of the peak [30]. The *APOE* e4 status was determined by genotyping 2 single nucleotide polymorphisms, rs42938 and rs7412, using TaqMan assays based on the ABI 7900HT fast real-time PCR system (Applied Biosystems Inc., Foster City, CA, USA [31]).

### Other outcomes

The study also assesses frailty status based on modifications of the criteria of Fried et al. [32]. Cognitive frailty is defined based on the consensus from the International Academy of Nutrition and Aging and the International Association of Gerontology and Geriatrics (IANA-IAGG) in 2013, and extended definitions were developed in our previous work based on various cognitive domains and physical and/or psychosocial frailty [33]. The TIGER data have been linked with the national death registry up to December 2021, and the data from the senior health check-up program at NTUH have been incorporated to obtain data from health check-ups. Additionally, healthcare utilization, including the number of hospitalizations and operations, is also collected.

## KEY FINDINGS

### Cognition, frailty, handgrip strength, and mortality

In 2020, we newly proposed extended definitions of cognitive frailty, exploring the combination of frailty dimensions (i.e., physical, psychosocial, and global frailty) and impaired cognitive domains. The difference between the 2013 definition of cognitive frailty by IANA-IAGG and our cognitive-global frailty is that our global frailty further includes “psychosocial frailty,” a component not included in the 2013 definition but crucial for the well-being of older adults. We found that cognitive-global frailty had a better predictive ability for all-cause mortality in older adults than the traditional definition proposed by the IANA-IAGG in 2013 [34]. Additionally, we found that physical frailty was associated with poor global cognition, memory, and executive function, while psychosocial frailty was associated with poor global cognition and attention [33]. In 2022, our findings showed that severe sarcopenia and poor grip strength were associated with both global and domain-specific cognitive impairment in older adults [35]. A subsequent 7-year study demonstrated that, males with reduced handgrip strength and handgrip strength asymmetry were associated with a higher risk of cognitive impairment across various domains, compare with females [36].

### Air pollutants and cognition

To investigate the impact of environmental exposure on cognitive function, we investigated and found that long-term exposure (1994-2017) to low-level air pollutants (below EPA standards) was associated with poor cognitive performance over a 4-year period [37]. In 2023, we newly explored the interaction between indoor air quality and low-level exposure to outdoor air pollutants, including PM_2.5_, PM_coarse_, NO_2_, O_3_, SO_2_, and CO [38]. This study also proposed novel multi-pollutant models that more accurately reflect real-world situations. Furthermore, a ventilation score was newly developed to assess indoor air quality based on daily indoor time and ventilation status, taking into account window/door openness and the use of air-circulation equipment during the day and night over 4 seasons. Our findings could potentially inform amendments to air quality standards and underscore the importance of the impact of indoor air quality on cognition.

### Lifestyle, diet, and cognition

Additionally, TIGER has investigated the impact of modifiable lifestyle factors on cognitive function. Our research demonstrated that 5 lifestyle factors, including high intake of vegetables and fish, regular exercise, not smoking, and light to moderate alcohol consumption, as well as 3 socioeconomic status indicators (high annual household income [> 33,333 US dollar], high occupational complexity, and high education level [> 12 years]), were significantly protective against cognitive decline [39]. Due to geographic differences in food resources and dietary habits, in 2017, we newly identified 3 dietary patterns (“vegetable,” “meat,” and “traditional”) among Chinese ethnic older adults. Our results suggested that the “vegetable” and “traditional” dietary patterns, which included fermented foods and pickled vegetables, protected against memory decline, while the “meat” dietary pattern increased the risk of verbal fluency decline [40]. In 2019, another study from TIGER indicated that a high-quality diet (assessed by the modified Alternative Healthy Eating Index based on the Dietary Guidelines for Americans and Food Patterns Equivalents database) was associated with a lower risk of global cognitive and attention decline over 2 years, and the results became more evident in participants with a high diversity of vegetable intake [41]. Furthermore, only a limited number of studies have repeatedly collected dietary data. In this elderly Asian population with longitudinal data, we were able to identify 3 distinct trajectories of dietary quality (“deteriorating,” “improving,” and “stable-high”) over 6 years [42]. Our findings showed that maintaining consistently high dietary quality was linked to better cognitive performance, emphasizing the critical role of promoting sound diet quality in older adults.

### *Helicobacter pylori*, retinal markers, and cognition

Our research team has also studied various biomarkers (e.g., retinal images, and immunoglobulin G (IgG) levels for *Helicobacter pylori* exposure) and disease patterns. A cross-sectional study from TIGER showed that the highest quartile of *H. pylori* IgG levels was associated with poor language and attention performance compared with the lowest quartile [43]. Additionally, our study newly found a U-shape association (i.e., thinning or thickening of GC-IPL) with poor global cognition and memory performance in non-demented older adults, suggesting that it may serve as a noninvasive preclinical predictor of dementia [24]. We further investigated the association between retinal vascular complexity, estimated by fractal dimension, and cognition. Our results suggest that a reduction in retinal vascular complexity, in either the right or left eye, is associated with varying degrees of impairment in global or domain-specific cognition [25]. These findings show that retinal markers could predict the risk of dementia in the preclinical phase.

### Renal function, olfactory function, and cognition

For research on kidney dysfunction and cognitive function, a cross-sectional study in TIGER showed that kidney dysfunction was associated with poor global cognition and lower frontal, partial, temporal, occipital, and insular lobar cerebral cortical thickness [22]. In 2020, a 4-year study from TIGER revealed that kidney dysfunction and cortical thinning jointly contributed to cognitive decline, especially in attention performance [23]. Another 4-year study found that odor identification deficits had an increased risk of poor global or domain-specific cognitive function in dementia-free older adults [44].

### Comorbidities, sleep, and cognition

In 2023, we identified sex-specific multimorbid patterns in older Taiwanese and found that they were differentially associated with poor cognitive performance in the presence of informative dropouts [18]. These multimorbid patterns (especially the “renal-vascular” pattern in males) were different from the patterns observed in Western countries, as renal diseases are more prevalent in Taiwan. Additionally, few studies have examined the relationship between subclinical depression and cognitive impairment, particularly MCI, while taking into account factors including sleep quality and daytime sleepiness. Our study investigated the interplay between these variables and found that good sleep quality, coupled with the absence of excessive daytime sleepiness, was associated with better memory performance over time. These findings highlight the significance of sleep and its relationship with preclinical depression and cognition among older adults [45].

## STRENGTHS AND WEAKNESSES

This study has several strengths. First, TIGER includes a comprehensive range of geriatric assessments and links the data with the national mortality registry and medical records from the senior health check-up program of NTUH. Although several epidemiological cohorts including community-dwelling older adults have been established in Taiwan [10-13], most have focused on general geriatric conditions, lacked data on domain-specific cognitive performance, and/or had irregular follow-up or cross-sectional data. Additionally, we used long-term (> 20 years) air pollutants data collected by monitoring stations of the Taiwan EPA to estimate individuals’ residential exposure to air pollutants in the study area (Taipei metropolis), which is better than regional exposure and exposure based on the area of clinic visit. Second, the biennially repeated measures of cognition and other variables, as well as blood sample collection every 4 years, provide valuable information on the temporal changes in health status, particularly cognitive performance and frailty, in older adults, in older adults. The data from TIGER allow us to fill in these gaps in the Taiwanese older population over the past decade, and make important comparisons with findings from Western countries. Third, to reduce dropout, we conducted telephone interviews for participants who were unable to attend a face-to-face interview during follow-ups. Therefore, the mean annual attrition rate in TIGER is 6.1%, which is lower than the average attrition rate of other cohorts including older adults [46]. This low attrition rate reflects the careful design and maintenance of this cohort study.

The study has some limitations. First, TIGER participants were recruited from a senior health check-up program in northern Taiwan (a metropolitan area with a higher educational level, i.e., only < 3% with years of education < 6 years), which may limit the generalizability of the findings to older populations in rural or suburban areas. However, the MCI prevalence in TIGER I (18.4%) is similar to that in a national survey during 2011-2013 [3], and the age-sex-adjusted incidence rate of suspected dementia (12.5 per 1,000 person-years) is close to that reported in a population-based study conducted with Asian Americans during 1999-2019 [47]. Participants who attended the health check-up program tended to be healthier than the general population at baseline. However, as the follow-up time increased, the participants’ health status declined and became more representative of the general population [48]. Second, the availability of repeated neuroimaging data was limited due to funding constraints and the willingness of participants to undergo re-assessment. Nonetheless, neuroimaging characteristics may change over time, and including repeated data on neuroimaging factors may be considered in future investigations to advance our understanding of the progression of cognitive impairment.

## DATA ACCESSIBILITY

We are looking forward to more collaborations with national and international studies via data sharing. Those interested in collaboration or data sharing are welcome to contact the corresponding authors: Yen-Ching Chen (e-mail: karenchen@ntu.edu.tw) and Jen-Hau Chen (e-mail: jhhchen@ntu.edu.tw). Further information about TIGER can be acquired by visiting the website (https://homepage.ntu.edu.tw/~karenchen/projects.html) or contacting the corresponding authors via e-mails.

## Figures and Tables

**Table 1.** Characteristics of TIGER I study participants by age groups at baseline (2011-2013, n=605)

Variables	Age (yr)				Total (n=605)
	65-69 (n=192)	70-74 (n=186)	75-79 (n=132)	≥80 (n=95)	
Education (yr)	13.9±3.2^*^	13.6±3.8^*^	12.4±4.5^*^	12.9±4.7^*^	13.3±4.0
BMI (kg/m^2^)	24.1±3.0	23.9±2.7	23.9±3.4	23.9±2.9	24.0±3.0
Physical activity (MET-min/wk)	1,847.9±1,348.6^*^	1,900.6±1,924.3^*^	1,535.7±1,391.8^*^	1,415.8±1,201.1^*^	1,728.1±1,546.3
Gait speed (8 feet; sec)	3.1±0.7^*^	3.3±1.1^*^	3.8±1.1^*^	4.0±1.3^*^	3.4±1.1
ADL	99.5±2.1	99.3±3.2	98.7±3.1	99.5±9.7	99.3±4.6
IADL	7.9±0.6^*^	7.8±0.5^*^	7.8±0.6^*^	7.6±1.1^*^	7.8±0.7
MoCA-T score	27.3±2.2^*^	26.4±2.9^*^	25.2±3.9^*^	23.9±4.2^*^	26±3.4
LM: immediate theme recall^1^	0.28±0.86^*^	0.10±0.93^*^	-0.19±1.04^*^	-0.51±1.10^*^	0±1.0
LM: immediate free recall^1^	0.38±0.94^*^	0.06±0.91^*^	-0.22±1.01^*^	-0.57±0.93^*^	0±1.0
LM: delayed theme recall^1^	0.30±0.85^*^	0.06±0.94^*^	-0.18±1.05^*^	-0.47±1.10^*^	0±1.0
LM: delayed free recall^1^	0.37±0.97^*^	0.04±0.92^*^	-0.21±1.01^*^	-0.52±0.91^*^	0±1.0
Digit span forward^1^	0.44±0.68^*^	0.10±0.86^*^	-0.33±1.14^*^	-0.65±1.11^*^	0±1.0
Digit span backward^1^	0.31±0.97^*^	0.02±0.98^*^	-0.24±0.95^*^	-0.36±0.97^*^	0±1.0
Trail Making Test- Part A^1^	0.41±0.56^*^	0.06±0.99^*^	-0.27±1.04^*^	-0.58±1.24^*^	0±1.0
Trail Making Test- Part B^1^	0.42±0.84^*^	0.14±0.90^*^	-0.31±0.98^*^	-0.71±1.02^*^	0±1.0
Verbal fluency tests^1^	0.42±0.90^*^	0.07±0.90^*^	-0.17±0.99^*^	-0.73±0.94^*^	0±1.0
Female	129 (67.2)^*^	96 (51.6)^*^	68 (51.5)^*^	33 (34.7)^*^	326 (53.9)
*APOE* e4 carriers	27 (14.1)	38 (20.7)	20 (15.3)	12 (12.6)	97 (16.1)
Annual disposable income >USD 33,333	85 (47.0)^*^	80 (45.7)^*^	47 (38.5)^*^	35 (38.9)^*^	247 (43.5)
Cigarette smoking	30 (15.6)	25 (13.4)	19 (14.4)	23 (24.2)	97 (16.0)
Alcohol consumption	37 (19.3)	38 (20.4)	24 (18.2)	24 (25.3)	123 (20.3)
Depressive symptoms	18 (9.4)	12 (6.5)	16 (12.1)	7 (7.4)	53 (8.8)
Hypertension	90 (46.9)^*^	116 (62.4)^*^	100 (75.8)^*^	70 (73.7)^*^	376 (62.2)
Hyperlipidemia	101 (52.6)	95 (51.1)	67 (50.8)	45 (47.4)	308 (50.9)
Diabetes mellitus	29 (15.1)	28 (15.1)	22 (16.7)	15 (15.8)	94 (15.5)

Values are presented as mean±standard deviation or number (%). TIGER, Taiwan Initiative for Geriatric Epidemiological Research; BMI, body mass index; MET, metabolic equivalent of task; ADL, activities of daily living; IADL, instrumental activities of daily living; MoCA-T, Taiwanese version of the Montreal Cognitive Assessment; LM, logical memory; *APOE*, apolipoprotein E; MCI, mild cognitive impairment; USD, US dollar.

1 To facilitate comparisons across different domain-specific cognitive tests, we standardized all cognitive test scores by Z transformation based on the mean and standard deviation at the baseline of each test; A higher standardized cognitive score in all neuropsychological measures indicated better performance.

* p<0.05.

**Table 2.** Data collection at each follow-up of TIGER I (2011-2024)

Data	Baseline (2011-2013)	Follow-up 1 (2013-2015)	Follow-up 2 (2015-2017)	Follow-up 3 (2017-2019)	Follow-up 4 (2019-2022)	Follow-up 5 (2022-2024)
Cognitive assessment						
Global cognition: MoCA-T	O	O	O	O	O	O
Memory domain: WMS-III logical memory	O	O	O	O	O	O
Executive function: Trail-making test	O	O	O	O	O	O
Attention domain: WMS-III Digit span test	O	O	O	O	O	O
Verbal fluency	O	O	O	O	O	O
Questionnaire						
Socio-demography and anthropometry	O	-	-	-	O	O
Medical history (disease and medication)	O	O	O	O	O	O
Physical activity: IPQA	O	O	O	O	O	O
Physical function: ADL, IADL	O	O	O	O	O	O
Depressive symptoms: CESD	O	O	O	O	O	O
Sleep assessment: PSQI, ESS	-	-	O	O	O	O
Stressful life events: SRRS	-	-	O	O	O	O
Dietary data: FFQ	O	-	O	O	O	O
Swallowing function: Eat-10	-	-	-	-	-	O
Hearing assessment: HHIE	-	-	-	-	-	O
Physical measures						
Body composition: BIA	O	O	O	O	O	O
Hand grip strength	-	-	O	O	O	O
Lower extremity function: SPPB	O	O	O	O	O	O
Ankle-brachial index	O	-	-	O	-	-
Intima-media thickness	O	-	-	-	-	-
Retinal imaging: fundus photography, OCT	-	-	O	-	-	O
Olfactory function: SSIT	-	-	O	-	O	O
Dental assessment	O	-	-	O	O	O
Brain images: MRI	O	-	-	-	-	-
Environmental exposure						
Air pollutants exposure	O	O	O	O	O	O
Indoor air quality	-	-	-	O	O	O
Biological specimens						
Serum markers, DNA	O	-	O	-	O	-
Metabolomics NMR (plasma)	O	-	-	-	-	-
Urine	-	-	-	-	O	-
Hair	-	-	O	-	-	-
Other outcomes of interests:						
Frailty	O	O	O	O	O	O
Mortality, health care utilization	-	O	O	O	O	O

Data collection in TIGER II (2019-present, n=629) is consistent with TIGER I from follow-up 4 and thereafter. TIGER, Taiwan Initiative for Geriatric Epidemiological Research; MoCA-T, Taiwanese version of the Montreal Cognitive Assessment; WMS, Wechsler Memory Scale; IPAQ, International Physical Activity Questionnaire; ADL, activities of daily living; IADL, instrumental activities of daily living; CSED, Center for Epidemiological Studies-Depression; PSQI, Pittsburgh Sleep Quality Index; ESS, Epworth Sleepiness Scale; SRRS, Social Readjustment Rating Scale; FFQ, food frequency questionnaire; HHIE, Hearing Handicap Inventory for the Elderly; BIA, bioelectrical impedance analysis; SPPB, Short Physical Performance Battery; OCT, optical coherence tomography; SSIT, Sniffin’ Sticks Identification Test; MRI, magnetic resonance imaging; DNA, deoxyribonucleic acid; NMR, nuclear magnetic resonance.

## References

[1] World Health Organization (2012). Dementia: a public health priority. https://www.who.int/publications/i/item/dementia-a-public-health-priority.

[2] World Health Organization (2023). Dementia. https://www.who.int/news-room/fact-sheets/detail/dementia.

[3] Sun Y, Lee HJ, Yang SC, Chen TF, Lin KN, Lin CC (2014). A nationwide survey of mild cognitive impairment and dementia, including very mild dementia, in Taiwan. PLoS One.

[4] Ministry of Health and Welfare, Taiwan (2021). Health statistics. https://dep.mohw.gov.tw/DOS/lp-4445-113.html.

[5] Livingston G, Huntley J, Sommerlad A, Ames D, Ballard C, Banerjee S (2020). Dementia prevention, intervention, and care: 2020 report of the Lancet Commission. Lancet.

[6] Kim KW, Park JH, Kim MH, Kim MD, Kim BJ, Kim SK (2011). A nationwide survey on the prevalence of dementia and mild cognitive impairment in South Korea. J Alzheimers Dis.

[7] Butler SM, Ashford JW, Snowdon DA (1996). Age, education, and changes in the Mini-Mental State Exam scores of older women: findings from the Nun Study. J Am Geriatr Soc.

[8] Ninomiya T, Nakaji S, Maeda T, Yamada M, Mimura M, Nakashima K (2020). Study design and baseline characteristics of a population-based prospective cohort study of dementia in Japan: the Japan Prospective Studies Collaboration for Aging and Dementia (JPSC-AD). Environ Health Prev Med.

[9] Petersen RC, Aisen PS, Beckett LA, Donohue MC, Gamst AC, Harvey DJ (2010). Alzheimer’s Disease Neuroimaging Initiative (ADNI): clinical characterization. Neurology.

[10] Health Promotion Administration (2015). 2011 Taiwan longitudinal study on aging survey report. https://www.hpa.gov.tw/Pages/Detail.aspx?nodeid=242&pid=1282.

[11] Hsu CC, Chang HY, Wu IC, Chen CC, Tsai HJ, Chiu YF (2017). Cohort profile: the healthy aging longitudinal study in Taiwan (HALST). Int J Epidemiol.

[12] Wu YH, Liu LK, Chen WT, Lee WJ, Peng LN, Wang PN (2015). Cognitive function in individuals with physical frailty but without dementia or cognitive complaints: results from the I-Lan Longitudinal Aging Study. J Am Med Dir Assoc.

[13] Wang MC, Li TC, Li CI, Liu CS, Lin CH, Lin WY (2020). Cognitive function and its transitions in predicting all-cause mortality among urban community-dwelling older adults. BMC Psychiatry.

[14] Dalton JE, Pederson SL, Blom BE, Holmes NR (1987). Diagnostic errors using the Short Portable Mental Status Questionnaire with a mixed clinical population. J Gerontol.

[15] Gluhm S, Goldstein J, Loc K, Colt A, Liew CV, Corey-Bloom J (2013). Cognitive performance on the mini-mental state examination and the montreal cognitive assessment across the healthy adult lifespan. Cogn Behav Neurol.

[16] Wang PN, Hong CJ, Lin KN, Liu HC, Chen WT (2011). APOE ε4 increases the risk of progression from amnestic mild cognitive impairment to Alzheimer’s disease among ethnic Chinese in Taiwan. J Neurol Neurosurg Psychiatry.

[17] Liu CK, Lai CL, Tai CT, Lin RT, Yen YY, Howng SL (1998). Incidence and subtypes of dementia in southern Taiwan: impact of socio-demographic factors. Neurology.

[18] Hsieh PI, Chen YC, Chen TF, Chiou JM, Chen JH (2023). Multimorbid patterns and cognitive performance in the presence of informative dropout among community-dwelling Taiwanese older adults. Innov Aging.

[19] Tsai CF, Lee WJ, Wang SJ, Shia BC, Nasreddine Z, Fuh JL (2012). Psychometrics of the Montreal Cognitive Assessment (MoCA) and its subscales: validation of the Taiwanese version of the MoCA and an item response theory analysis. Int Psychogeriatr.

[20] Shah S, Vanclay F, Cooper B (1989). Improving the sensitivity of the Barthel Index for stroke rehabilitation. J Clin Epidemiol.

[21] Slot RE, Verfaillie SC, Overbeek JM, Timmers T, Wesselman LM, Teunissen CE (2018). Subjective Cognitive Impairment Cohort (SCIENCe): study design and first results. Alzheimers Res Ther.

[22] Chen CH, Chen YF, Chiu MJ, Chen TF, Tsai PH, Chen JH (2017). Effect of kidney dysfunction on cerebral cortical thinning in elderly population. Sci Rep.

[23] Chen CH, Chen YF, Tsai PH, Chiou JM, Lai LC, Chen TF (2020). Impacts of kidney dysfunction and cerebral cortical thinning on cognitive change in elderly population. J Alzheimers Dis.

[24] Liu YL, Hsieh YT, Chen TF, Chiou JM, Tsai MK, Chen JH (2018). Retinal ganglion cell-inner plexiform layer thickness is nonlinearly associated with cognitive impairment in the community-dwelling elderly. Alzheimers Dement (Amst).

[25] Wu TY, Hsieh YT, Wang YH, Chiou JM, Chen TF, Lai LC (2023). The association between retinal vascular fractal dimension and cognitive function in the community-dwelling older adults cohort TIGER. J Formos Med Assoc.

[26] Aboyans V, Criqui MH, Abraham P, Allison MA, Creager MA, Diehm C (2012). Measurement and interpretation of the anklebrachial index: a scientific statement from the American Heart Association. Circulation.

[27] Hummel T, Sekinger B, Wolf SR, Pauli E, Kobal G (1997). ‘Sniffin’ sticks’: olfactory performance assessed by the combined testing of odor identification, odor discrimination and olfactory threshold. Chem Senses.

[28] Lee MS, Pan WH, Liu KL, Yu MS (2006). Reproducibility and validity of a Chinese food frequency questionnaire used in Taiwan. Asia Pac J Clin Nutr.

[29] Christakos G, Serre ML (2000). BME analysis of spatiotemporal particulate matter distributions in North Carolina. Atmos Environ.

[30] You YS, Lin CY, Liang HJ, Lee SH, Tsai KS, Chiou JM (2014). Association between the metabolome and low bone mineral density in Taiwanese women determined by (1)H NMR spectroscopy. J Bone Miner Res.

[31] Ghebranious N, Ivacic L, Mallum J, Dokken C (2005). Detection of ApoE E2, E3 and E4 alleles using MALDI-TOF mass spectrometry and the homogeneous mass-extend technology. Nucleic Acids Res.

[32] Fried LP, Tangen CM, Walston J, Newman AB, Hirsch C, Gottdiener J (2001). Frailty in older adults: evidence for a phenotype. J Gerontol A Biol Sci Med Sci.

[33] Chen JH, Shih HS, Tu J, Chiou JM, Chang SH, Hsu WL (2021). A longitudinal study on the association of interrelated factors among frailty dimensions, cognitive domains, cognitive frailty, and all-cause mortality. J Alzheimers Dis.

[34] Kelaiditi E, Cesari M, Canevelli M, van Kan GA, Ousset PJ, Gillette-Guyonnet S (2013). Cognitive frailty: rational and definition from an (I.A.N.A./I.A.G.G.) international consensus group. J Nutr Health Aging.

[35] Peng TC, Chiou JM, Chen TF, Chen YC, Chen JH (2023). Grip strength and sarcopenia predict 2-year cognitive impairment in community-dwelling older adults. J Am Med Dir Assoc.

[36] Peng TC, Chiou JM, Chen YC, Chen JH (2024). Handgrip strength asymmetry and cognitive impairment risk: insights from a seven-year prospective cohort study. J Nutr Health Aging.

[37] ddChen JH, Kuo TY, Yu HL, Wu C, Yeh SL, Chiou JM (2020). Long-term exposure to air pollutants and cognitive function in Taiwanese community-dwelling older adults: a four-year cohort study. J Alzheimers Dis.

[38] Chen YC, Hsieh PI, Chen JK, Kuo E, Yu HL, Chiou JM (2023). Effect of indoor air quality on the association of long-term exposure to low-level air pollutants with cognition in older adults. Environ Res.

[39] Weng PH, Chen JH, Chiou JM, Tu YK, Chen TF, Chiu MJ (2018). The effect of lifestyle on late-life cognitive change under different socioeconomic status. PLoS One.

[40] Chen YC, Jung CC, Chen JH, Chiou JM, Chen TF, Chen YF (2017). Association of dietary patterns with global and domain-specific cognitive decline in Chinese elderly. J Am Geriatr Soc.

[41] Chou YC, Lee MS, Chiou JM, Chen TF, Chen YC, Chen JH (2019). Association of diet quality and vegetable variety with the risk of cognitive decline in Chinese older adults. Nutrients.

[42] Chen LW, Chou YC, Lee MS, Chiou JM, Chen JH, Chen YC (2023). Longitudinal trajectories of dietary quality and cognitive performance in older adults: results from a 6-year cohort study. Clin Nutr.

[43] Han ML, Chen JH, Tsai MK, Liou JM, Chiou JM, Chiu MJ (2018). Association between Helicobacter pylori infection and cognitive impairment in the elderly. J Formos Med Assoc.

[44] Wang MC, Chiou JM, Chen YC, Chen JH (2023). Association between olfactory dysfunction and cognitive impairment in dementia-free older adults: a prospective cohort study in Taiwan. J Alzheimers Dis.

[45] Hsieh CJ, Chiou JM, Chen TF, Chen YC, Chen JH (2023). Association of subclinical depressive symptoms and sleep with cognition in the community-dwelling older adults. J Formos Med Assoc.

[46] Chatfield MD, Brayne CE, Matthews FE (2005). A systematic literature review of attrition between waves in longitudinal studies in the elderly shows a consistent pattern of dropout between differing studies. J Clin Epidemiol.

[47] Kornblith E, Bahorik A, Boscardin WJ, Xia F, Barnes DE, Yaffe K (2022). Association of race and ethnicity with incidence of dementia among older adults. JAMA.

[48] Strotmeyer ES, Arnold AM, Boudreau RM, Ives DG, Cushman M, Robbins JA (2010). Long-term retention of older adults in the Cardiovascular Health Study: implications for studies of the oldest old. J Am Geriatr Soc.

